# Contribution of Fdh3 and Glr1 to Glutathione Redox State, Stress Adaptation and Virulence in *Candida albicans*

**DOI:** 10.1371/journal.pone.0126940

**Published:** 2015-06-03

**Authors:** Anna T. Tillmann, Karin Strijbis, Gary Cameron, Elahe Radmaneshfar, Marco Thiel, Carol A. Munro, Donna M. MacCallum, Ben Distel, Neil A. R. Gow, Alistair J. P. Brown

**Affiliations:** 1 Aberdeen Fungal Group, School of Medical Sciences, Institute of Medical Sciences, University of Aberdeen, Aberdeen, United Kingdom; 2 Department of Medical Biochemistry, Academic Medical Center, University of Amsterdam, Amsterdam, Netherlands; 3 Division of Applied Medicine, Mass Spectrometry Section, University of Aberdeen, Aberdeen, United Kingdom; 4 Institute for Complex Systems and Mathematical Biology, SUPA, University of Aberdeen, Aberdeen, United Kingdom; Louisiana State University, UNITED STATES

## Abstract

The major fungal pathogen of humans, *Candida albicans*, is exposed to reactive nitrogen and oxygen species following phagocytosis by host immune cells. In response to these toxins, this fungus activates potent anti-stress responses that include scavenging of reactive nitrosative and oxidative species via the glutathione system. Here we examine the differential roles of two glutathione recycling enzymes in redox homeostasis, stress adaptation and virulence in *C*. *albicans*: glutathione reductase (Glr1) and the S-nitrosoglutathione reductase (GSNOR), Fdh3. We show that the NADPH-dependent Glr1 recycles GSSG to GSH, is induced in response to oxidative stress and is required for resistance to macrophage killing. *GLR1* deletion increases the sensitivity of *C*. *albicans* cells to H_2_O_2_, but not to formaldehyde or NO. In contrast, Fdh3 detoxifies GSNO to GSSG and NH_3_, and *FDH3* inactivation delays NO adaptation and increases NO sensitivity. *C*. *albicans fdh3*⎔ cells are also sensitive to formaldehyde, suggesting that Fdh3 also contributes to formaldehyde detoxification. *FDH3* is induced in response to nitrosative, oxidative and formaldehyde stress, and *fdh3*Δ cells are more sensitive to killing by macrophages. Both Glr1 and Fdh3 contribute to virulence in the *Galleria mellonella* and mouse models of systemic infection. We conclude that Glr1 and Fdh3 play differential roles during the adaptation of *C*. *albicans* cells to oxidative, nitrosative and formaldehyde stress, and hence during the colonisation of the host. Our findings emphasise the importance of the glutathione system and the maintenance of intracellular redox homeostasis in this major pathogen.

## Introduction

The major fungal pathogen, *Candida albicans*, has the capacity to colonise diverse niches in its human host. This fungus is part of the microflora of the skin, mouth, gut and urogenital tracts of humans. *C*. *albicans* is a frequent cause of mucosal infections (thrush) in otherwise healthy individuals, with most women suffering at least one episode of vaginitis in their lifetime. *C*. *albicans* is also the most common fungal species responsible for life-threatening hospital-acquired bloodstream infections in immunocompromised patients [1–3].

The ability of *C*. *albicans* to thrive in diverse niches is dependent upon its robust adaptive responses to the local environmental stresses encountered in these niches. For example, immune cells such as macrophages combat microbial infection by exposing invading microbes to a battery of insults that include reactive nitrogen species (RNS), reactive oxygen species (ROS) and cationic fluxes [4–6]. Macrophages have been reported to generate up to 57 μM nitric oxide [7] and up to 14 mM hydrogen peroxide (H_2_O_2_) [8,9], although estimating the levels of specific ROS species during the oxidative burst is challenging [10,11]. Cation concentrations are around 150 mM in human blood, have been reported reach 0.2–0.3 M in phagocytes [5] and can increase to 600 mM in the kidney [12]. Therefore, *C*. *albicans* cells are exposed to significant oxidative, nitrosative and cationic stresses during host colonization and invasion [13–15].

Glutathione (L-γ-glutamylcysteinylglycine; GSH), the most abundant non-protein thiol in eukaryotic cells, plays a major role in protective responses to oxidative and nitrosative stress. Glutathione reacts with reactive oxygen and nitrogen species to generate glutathione adducts, such as glutathione disulphide (GSSG) and S-nitrosoglutathione (GSNO). In addition to detoxifying xenobiotics and free radicals, glutathione functions as a co-factor in many enzymatic reactions and is involved in amino acid transport and signalling [16,17]. S-glutathionylation protects protein thiols from irreversible over-oxidation [18]. Glutathione maintains intracellular redox homeostasis through the oxidation of its cysteine sulphydryl moiety and disulphide bond formation [19]. The low redox potential of glutathione (E = -240 mV), combined with its high intracellular concentration (1–10 mM), contribute to its large redox buffering capacity [20,21]. Fungal genes involved in both glutathione synthesis and the recycling of GSSG and GSNO are tightly regulated in response to stress exposure [21,22].

Glutathione is synthesised in two ATP-dependent steps. In the first rate-limiting step, γ-glutamylcysteine synthetase (Gsh1 in *S*. *cerevisiae*) ligates glutamate and cysteine to form the dipeptide γ-glutamylcysteine [23]. In the second enzymatic step glutathione synthetase (Gsh2 in *S*. *cerevisiae*) converts γ-glutamylcysteine and glycine into glutathione [24]. In *S*. *cerevisiae*, Gsh1 catalyses the rate-limiting step in GSH synthesis and *GSH1* deletion significantly increases the intracellular redox potential to -178 mV, compared with -235 mV in wild type cells [25]. Consequently, *S*. *cerevisiae gsh1* cells are sensitive to oxidative stress. This mutant also displays growth defects on minimal medium in the absence of stress [23], a phenotype that can be suppressed by the addition of exogenous GSH [26]. These results indicate that GSH is an essential metabolite for the growth of *S*. *cerevisiae*. Exogenous GSH also confers protection against oxidative stresses mediated by disulfiram [27], hypochlorite, chlorite [28] and heavy metals [29]. In *C*. *albicans*, the deletion of *GCS1* (the homologue of *S*. *cerevisiae GSH1*) causes cells to upregulate typical apoptotic markers, reduces their resistance to killing by human macrophages and decreases their virulence in a murine model of disseminated candidiasis [30], [31]. These observations highlight the importance of glutathione in general and for *C*. *albicans* pathogenicity in particular.

Following the formation of glutathione adducts, such as GSSG and GSNO, these molecules are recycled to reform glutathione via glutathione reductase and S-nitrosoglutathione reductase (GSNOR). In *S*. *cerevisiae*, glutathione reductase (Glr1), a member of the FAD-containing pyridine disuphide oxidoreductase family, is required for protection against oxidative stress [32]. It reduces GSSG in an NADPH-dependent manner following the reaction: GSSG + NADPH + H^+^ → 2GSH + NADP^+^. Thus while detoxifying GSSG, Glr1 concomitantly lowers the intracellular NADPH/NADP^+^ ratio [33]. Glr1 is not essential in *S*. *cerevisiae* since thioredoxins, Trx1 and Trx2, can detoxify GSSG to GSH in the absence of Glr1 [34]. Indeed, *Drosophila* and trypanosomes do not encode glutathione reductases, but detoxify GSSG via the thioredoxin or trypanothione systems, respectively [35,36].

Glutathione-dependent formaldehyde reductases (GSNORs) are conserved from bacteria to humans [21,37], suggesting that GSNORs perform important functions in all living organisms. These enzymes not only regulate GSNO levels, but are also involved in the repair of S-nitrosylated proteins [37]. Consequently, *S*. *cerevisiae* cells lacking GSNOR (the *sfa1* mutant) display slow adaptation to nitrosative stress, but are not sensitive to oxidative stress [37]. GSNORs are GSH-dependent bi-functional enzymes that are able to reduce GSNO to form GSSG plus NH_3_, as well as detoxifying formaldehyde [37]. In plants, GSNOR modulates the extent of cellular *S*-nitrosothiol (SNO) formation following nitrosative stress and is required for disease resistance [38].

Despite the importance of glutathione and redox homeostasis for oxidative stress resistance and virulence in *C*. *albicans*, the relative contributions of Glr1 and GSNOR in glutathione recycling have not been examined in this fungus. Indeed, a GSNOR has not previously been identified in *C*. *albicans*. Therefore, we have examined the roles of Glr1 and GSNOR in *C*. *albicans*. We show that these enzymes are crucial for the maintainance of redox homeostasis in *C*. *albicans* and that they contribute to the virulence of this major fungal pathogen.

## Results

### The *C*. *albicans* genome encodes a putative glutathione reductase and an S-nitrosoglutathione reductase

**Fig 1:**
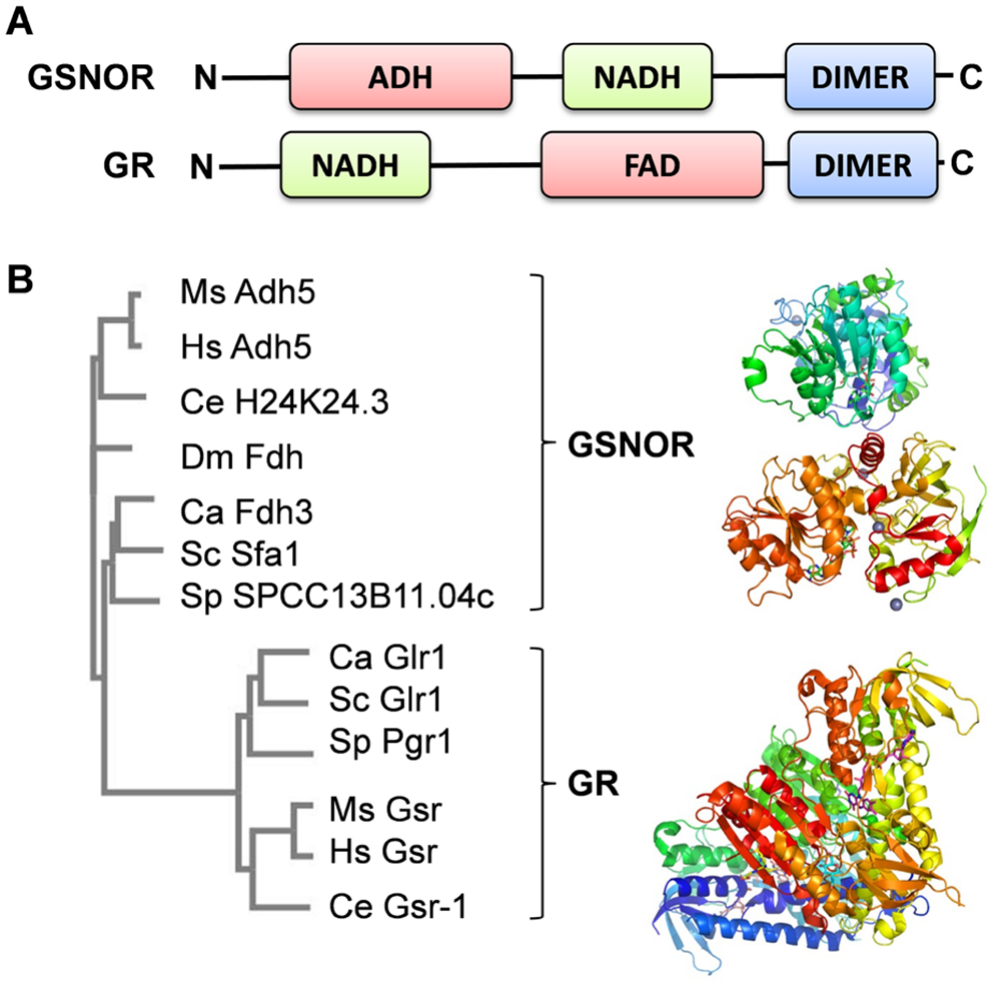
*C*. *albicans* Glr1 and Fdh3 belong to evolutionary conserved families of glutathione reductases (GRs) and S-nitroso-glutathione reductases (GSNORs), respectively. **(A)** The functional domains of GSH-dependent formaldehyde dehydrogenases class III (GSNORs) and NADPH-dependent glutathione reductases (GRs). GSNORs harbour a catalytic domain (ADH), an NAD(H) binding domain, and a dimerization domain. GRs have an NADH- and FAD-binding domains and a dimerization domain. **(B)** Phylogenetic tree of GSNOR- and GR-related proteins generated using ClustalW: homologs are presented from *Candida albicans* (CaFdh3, CaGlr1), *Saccharomyces cerevisiae* (ScSfa1, ScGlr1), *Schizosaccharomyces pombe* (SpSPCC13B11.04c, SpPgr1), *Mus musculus* (MsAdh5; MsGsr1), *Homo sapiens* (HsAdh5; HsGsr), *Drosophila melanogaster* (DmFdh) and *Caenorhabditis elegans* (CeH24K24.3; CeGsr-1). Structures are presented for human liver ChiChi alcohol dehydrogenase (protein data bank (pdb) accession code 1TEH; a GSNOR that has 65% sequence identity to CaFdh3p), and *S*. *cerevisiae* Glr1 (pdb accession code 2HQM; a GR with 66% sequence identity to CaGlr1p). Structure representations were made with PyMOL (http://www.pymol.org).

A single glutathione reductase gene has been annotated in the *C*. *albicans* genome on the basis of its sequence similarity to glutathione reductases (GRs) from other species [39]. *C*. *albicans GLR1* (C5_01520C) encodes a highly conserved NADPH-dependent glutathione reductase (S1 Fig) [40] that carries the classical NADH and FAD binding domains and dimerization domain of GRs (Fig 1A). Phylogenetically, *C*. *albicans* Glr1 is most closely related to GRs from other fungi, displaying 66% amino acid sequence identity to *S*. *cerevisiae* Glr1 (Fig 1B). Fungal GRs cluster with human, mouse and *Caenorhabditis elegans* GRs, clearly separable from GSNORs from the same organisms (Fig 1B).

*C*. *albicans* also contains a single GSNOR gene, *FDH3* (CR_10250C_A) [39]. Based on its sequence similarity to GSNORs from other organisms (S2 Fig), *FDH3* appears to encode a GSH-dependent formaldehyde dehydrogenase class III that contains alcohol dehydrogenase-like, NADH binding and dimerization domains (Fig 1A). GSNORs are highly conserved from bacteria to man [21]. Indeed, the sequence of *C*. *albicans* Fdh3 is 65% identical to human liver ChiChi alcohol dehydrogenase (Hs Adh5) (Fig 1B), which is a GSH-dependent formaldehyde dehydrogenase.

### *C*. *albicans* Fdh3 and Glr1 play differential roles in conferring formaldehyde, nitrosative and oxidative stress resistance

**Fig 2:**
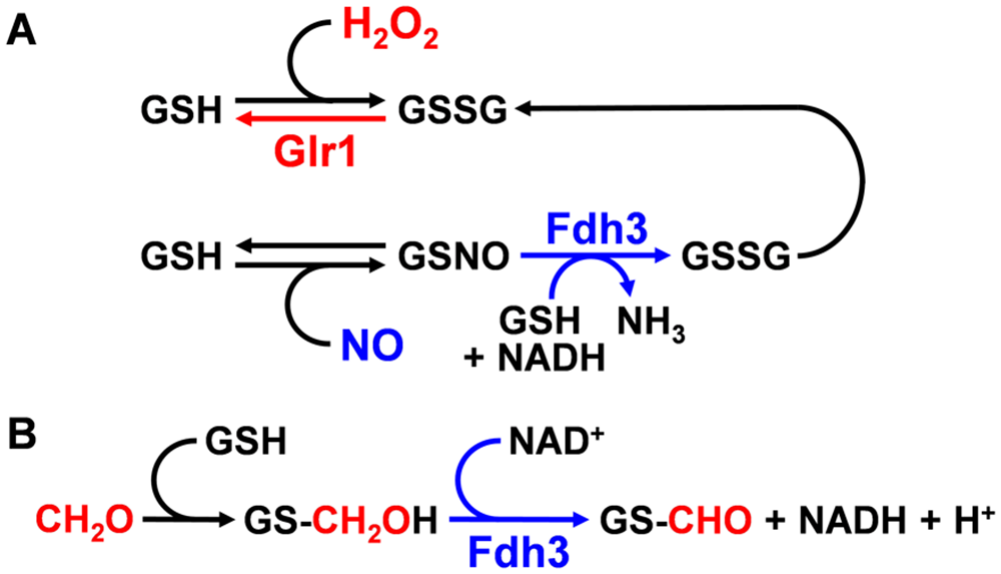
Predicted roles for Fdh3 and Glr1 in *C. albicans*. **(A)** Predicted roles for Fdh3 and Glr1 in GSNO and GSSG detoxification. **A** shows the working hypothesis of the major enzymes involved in the detoxification of GSSG (glutathione disulphide) and GSNO (S-nitrosoglutathione). When glutathione (GSH) is oxidised via H_2_O_2_ to GSSG, GSSG can be reduced with the help of the NADPH-dependent glutathione reductase (GR). We predict that the glutathione reductase of *Candida albicans* is *GLR1*. When GSH is exposed to NO, GSH is S-nitrosylated to GSNO. We predict that the S-nitrosoglutathione reductase (GSNOR) of *Candida albicans* is *FDH3*. **(B)** Predicted role for Fdh3 in formaldehyde detoxification. **B** shows the second enzymatic function of GSNOR the detoxification of formaldehyde. Formaldehyde reacts with glutathione (GSH) to form S-(hydroxmethyl)glutathione which then gets converted by Fdh3 and NAD+ to S-(formyl)glutathione.

GSNOR is the only enzyme so far characterised that enzymatically detoxifies GSNO [21], and both GSNOR and GR are critical for the maintenance of the glutathione redox balance under nitrosative and oxidative stress [21]. In *C*. *albicans*, Glr1 is presumed to act as a GR [39], and we reasoned that Fdh3 might act as the GSNOR, detoxifying GSNO and catalysing the detoxification of formaldehyde (CH_2_O) to formate (CHOO^-^) (Fig 2).

**Fig 3:**
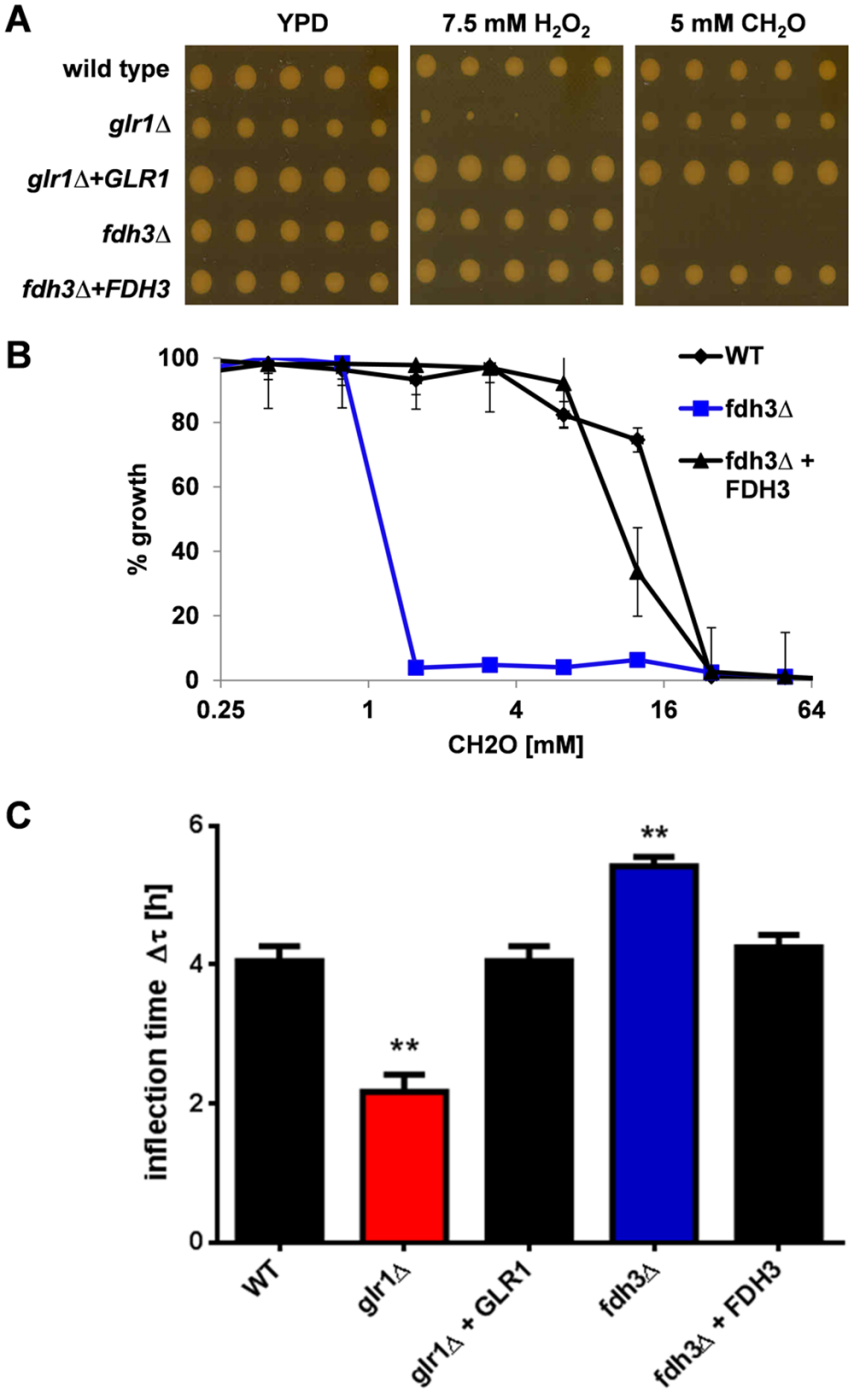
Differential sensitivities of *C*. *albicans fdh3*Δ and *glr1*Δ cells to hydrogen peroxide, nitric oxide and formaldehyde. **(A)** Sensitivity to hydrogen peroxide (7.5 mM H_2_O_2_) and formaldehyde (5 mM CH_2_O): wild type (CPK05); *glr1*Δ (CKS10), *glr1*Δ+*GLR1* (CKS31), *fdh3*Δ (ATT1); *fdh3*Δ+*FDH3* (ATT4) (Table 1). **(B)** Dose-dependent sensitivity to formaldehyde: wild type (CPK05); *fdh3*Δ (ATT1); *fdh3*Δ+*FDH3* (ATT4). **(C)** Differences in adaptation (inflection) time after nitrosative stress (2.5 mM DPTA NONOate): wild type (CPK05); *glr1*Δ (CKS10); *glr1*Δ+*GLR1* (CKS31); *fdh3*Δ (ATT1); *fdh3*Δ+*FDH3* (ATT4).

To test these predictions we constructed *C*. *albicans glr1*Δ and *fdh3*Δ null mutants, and then reintegrated wild type *GLR1* and *FDH3* genes to generate isogenic control strains. As predicted, the *glr1*Δ mutant was sensitive to hydrogen peroxide (7.5 mM), a phenotype that was suppressed by reintegration of *GLR1*. The *glr1*Δ mutant was resistant to formaldehyde (5 mM) (Fig 3A). In contrast, the *fdh3*Δ mutant was resistant to hydrogen peroxide, but sensitive to formaldehyde (Fig 3A and 3B). Reintegration of *FDH3* suppressed this sensitivity, confirming that Fdh3 is critical for formaldehyde resistance. These observations were consistent with the predicted differential roles for Fdh3 and Glr1 in the detoxification of formaldehyde and peroxide-induced GSSG, respectively.

We also investigated the impact of inactivating Fdh3 or Glr1 on the resistance of *C*. *albicans* cells to nitrosative stress (2.5 mM DPTA NONOate). As predicted (Fig 2), *fdh3*Δ cells were relatively sensitive to nitrosative stress, taking significantly longer than wild type cells to adapt to this stress (Fig 3C). Taken together, our data are consistent with the idea that the Fdh3 is a bi-functional enzyme involved in the detoxification of both formaldehyde and GSNO.

**Fig 4:**
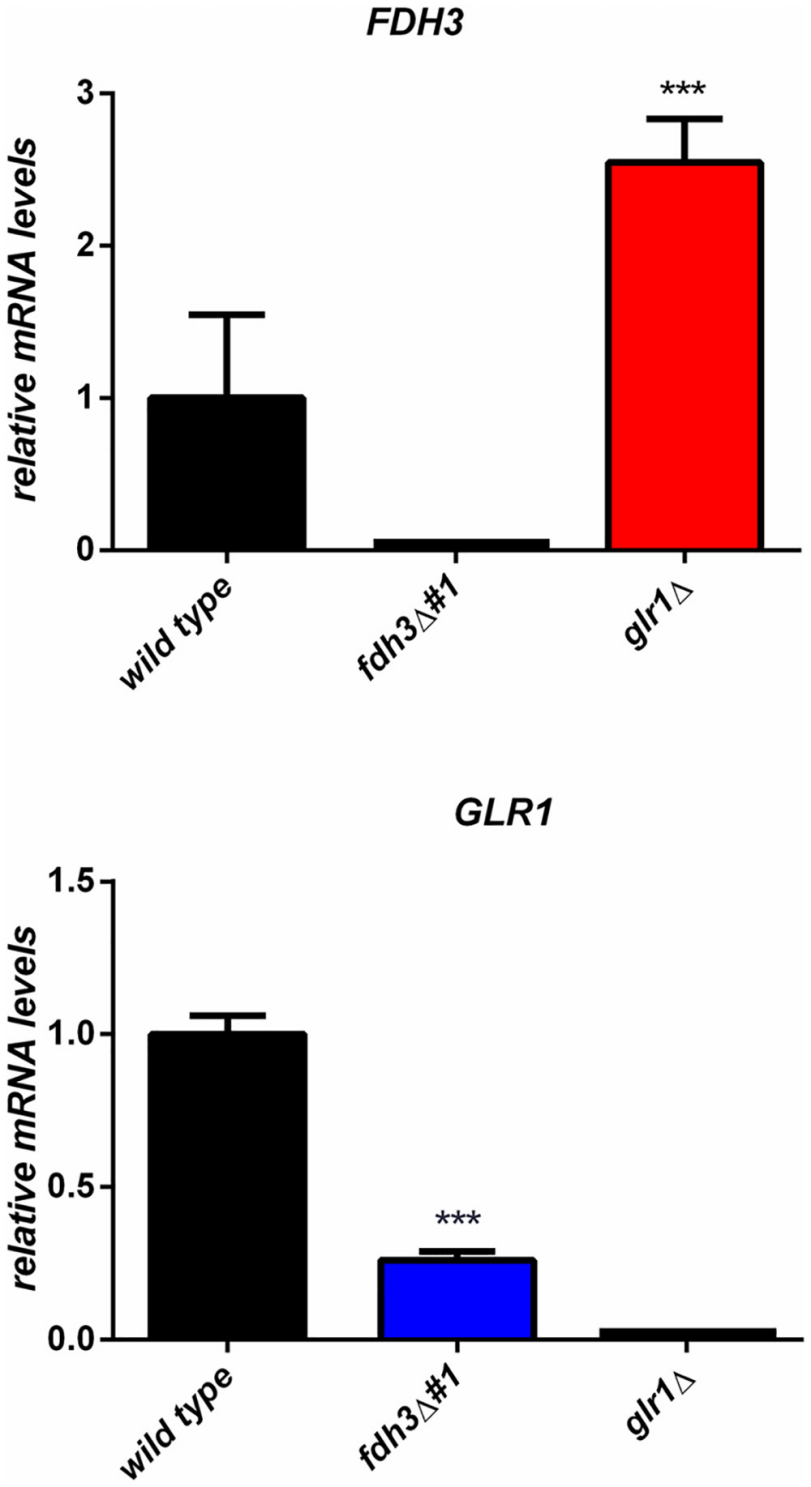
Effect of *FDH3* and *GLR1* deletion on basal gene expression. Quantification of *FDH3* and *GLR1* mRNA levels by qRT-PCR, relative to the internal *ACT1* mRNA control and normalised to wild type cells: wild type (BWP17); *fdh3*Δ (ATT1); *glr1*Δ (CKS10).

Surprisingly, *glr1*Δ cells were relatively resistant to nitrosative stress, adapting more quickly to DPTA NONOate than the wild type and *GLR1* reintegrant control strains (Fig 3C). To examine the basis for this we tested the impact of *GLR1* or *FDH3* deletion upon the expression of each mRNA by qRT-PCR (Fig 4). As expected, the *GLR1* and *FDH3* mRNAs were not detectable in their respective mutant strains. *GLR1* mRNA levels were reduced about 4-fold in *fdh3*Δ cells, relative to the wild type control. However, *FDH3* mRNA levels were elevated about 2.5-fold in *glr1*Δ cells (Fig 4). This increased *FDH3* expression could account for the increased nitrosative stress resistance of the *glr1*Δ strain (Fig 3C).

### Impact of Fdh3 and Glr1 deletion upon gene expression

**Fig 5:**
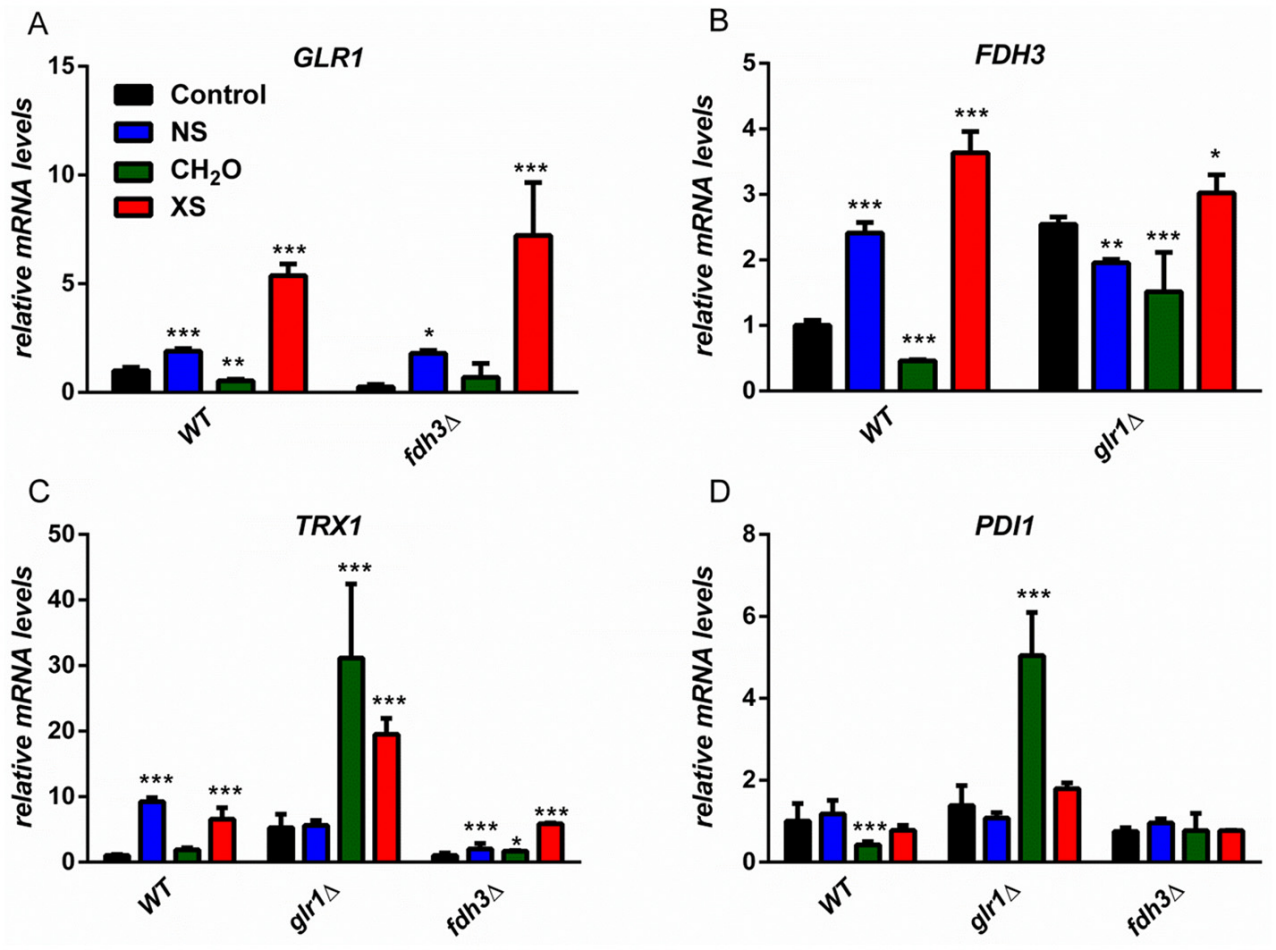
Impact of *FDH3* and *GLR1* deletion on transcript levels in respose to formaldehyde, oxidative or nitrosative stress. Transcript levels were quantified by qRT-PCR, relative to the internal *ACT1* mRNA control after 10 min of stress treatment and normalised to untreated wild type cells: wild type (CPK05); *fdh3*Δ (ATT1); *glr1*Δ (CKS10). Stresses were 2.5 mM CySNO (NS), 5 mM CH_2_O or 5 mM H_2_O_2_ (XS). Gene expression was assayed for the following genes: (A) *GLR1*, (B) *FDH3*, (C) *TRX1*, (D) *PDI1*.

The deletion of either *FDH3* or *GLR1* affects the expression of the other gene under basal conditions in the absence of stress (Fig 4). Therefore, we reasoned that the expression of these genes might also be perturbed in *glr1*Δ and *fdh3*Δ cells following exposure to oxidative, and nitrosative or formaldehyde stress. To test this we measured the expression levels of selected genes involved in stress defence by qRT-PCR, relative to the *ACT1* mRNA internal control, following a 10 min exposure to 5 mM H_2_O_2_, 2.5 mM CySNO or 5 mM formaldehyde. *GLR1* is known to be induced in response to oxidative stress [41], and therefore we examined its expression in response to these stresses in wild type and *fdh3*Δ cells (Fig 5A). The *GLR1* mRNA was up-regulated in response to oxidative and nitrosative stress, but was down-regulated following formaldehyde stress. Deletion of *FDH3* had a minimal effect upon this expression profile, except that *GLR1* expression was no longer reduced in response to formaldehyde stress. *FDH3* displayed a similar expression profile to *GLR1* in wild type cells (Fig 5B). *FDH3* was up-regulated following exposure to both oxidative and nitrosative stress, and down-regulated following formaldehyde stress. This regulation was perturbed in *glr1*Δ cells, primarily because basal *FDH3* mRNA levels were increased in the absence of stress, which was consistent with our previous observations (Fig 4).

We also examined the mRNA levels for *TRX1* (thioredoxin) under equivalent conditions (Fig 5C). As reported previously [41–43], *TRX1* was induced in response to oxidative and nitrosative stress. Interestingly, the response to oxidative stress was unaffected in *fdh3*Δ cells, but the response to nitrosative stress was attenuated. This is consistent with the idea that in the absence of Fdh3, GSSG generation might be reduced following nitrosative stress (Fig 2). In *glr1*Δ cells, but not in *fdh3*Δ cells, *TRX1* was strongly induced in response to formaldehyde stress (Fig 5C). This supports the hypothesis that intracellular GSSG levels might accumulate following formaldehyde stress in cells that lack Glr1, but contain Fdh3 (Fig 2).

Protein disulphide-isomerase is involved in redox-dependent protein folding, and therefore we also investigated *PDI1* mRNA levels under conditions where GSSG levels probably rise significantly (Fig 5D). Consistent with results found for *TRX1*, *PDI1* expression was strongly induced in *glr1*Δ cells, but not in *fdh3*Δ cells, after formaldehyde exposure.

### Effect of Glr1 and Fdh3 inactivation upon GSSG and GSNO detoxification

The data obtained are consistent with the predicted roles for Glr1 and Fdh3 in GSSG and GSNO detoxification (Fig 2). We then tested these predictions directly by examining the impact of deleting *GLR1* or *FDH3* upon GSSG and GSNO detoxification rates.

**Fig 6:**
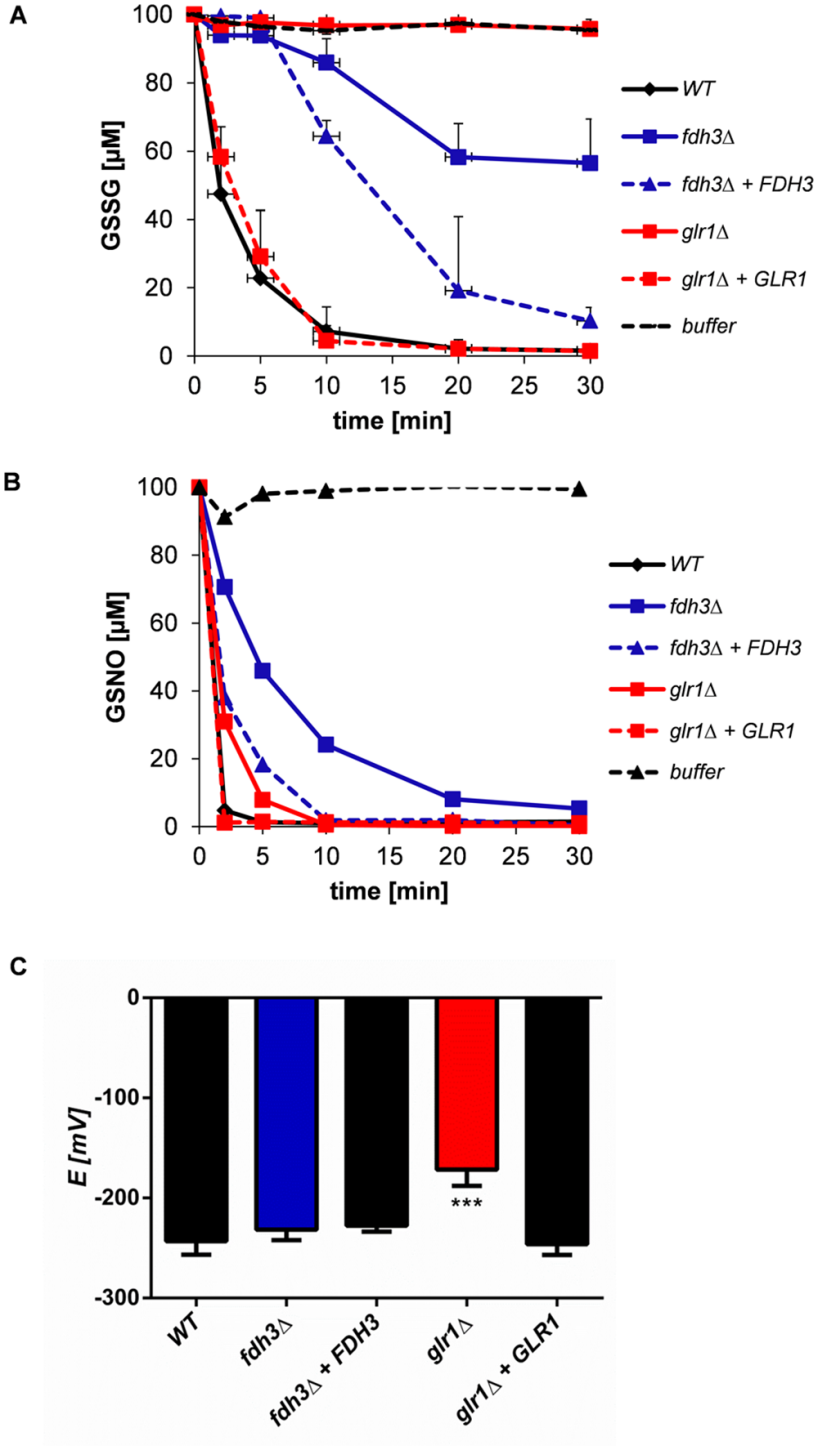
Lack of Fdh3 or Glr1 affects GSSG and GSNO detoxification and the glutathione redox potential. **(A)** GSSG detoxification by protein extracts from wild type (CPK05), *glr1*Δ (CKS10), *glr1*Δ+*GLR1* (CKS31), *fdh3*Δ (ATT1) and *fdh3*Δ+*FDH3* (ATT4) cells. **(B)** GSNO detoxification by protein extracts from wild type (CPK05), *glr1*Δ (CKS10), *glr1*Δ+*GLR1* (CKS31), *fdh3*Δ (ATT1) and *fdh3*Δ+*FDH3* (ATT4) cells. **(C)** Glutathione redox potential for wild type (CPK05), *glr1*Δ (CKS10), *glr1*Δ+GLR1 (CKS31), *fdh3*Δ (ATT1) and *fdh3*Δ+*FDH3* (ATT4) strains.

GSSG detoxification was assayed by LC-MS/MS. GSSG detoxification rates were minimal in the absence of protein extract (buffer alone), but the majority of GSSG was detoxified within 10 min by wild type protein extracts (Fig 6A). Deletion of *GLR1* completely blocked GSSG detoxification rates, and these rates were restored by reintegration of the wild type *GLR1* gene. GSSG detoxification rates were also reduced in *fdh3*Δ cells, which could be due to the 4-fold reduction in *GLR1* expression in these cells (Fig 4). We conclude that *GLR1* is essential for rapid GSSG detoxification in *C*. *albicans*, which is consistent with the view that this gene does indeed encode a glutathione reductase.

GSNO detoxification was also assayed in wild-type and mutant cells (Fig 6B). As expected (Fig 2), *GLR1* inactivation had no major effect upon GSNO detoxification, whereas rates of GSNO detoxification were reduced in *fdh3*Δ cells (Fig 6B). However, GSNO detoxification was not completely blocked following Fdh3 deletion, suggesting that other *C*. *albicans* enzymes, in addition to Fdh3, must have denitrosylation or trans-nitrosylation activity. The identity of these enzymes remains to be determined.

Given the impact of Glr1 inactivation upon GSSG detoxification in particular, the *glr1*Δ mutation is likely to perturb redox homeostasis in *C*. *albicans* cells. The GSSG/GSH redox potential (*ΔE*), which essentially reflects the relative amounts of reduced versus oxidized glutathione, was measured in mutant and wild type cells (Fig 6C). The GSSG/GSH redox potential of *C*. *albicans fdh3*Δ cells displayed no significant difference from wild type or *FDH3* reintegrant controls, despite the reduced ability of this mutant to detoxify GSSG (Fig 6A). However, the GSSG/GSH redox potential of *glr1*Δ cells was significantly elevated, which correlated with their inability to detoxify GSSG (Fig 6A and 6C). Indeed, the redox potential of *glr1*Δ cells was close to the value of -180 mV, which has been associated with cell death in other systems [20,44]. This was consistent with the relatively slow growth of *glr1*Δ colonies (Fig 1A).

### Both *FDH3* and *GLR1* influence *C*. *albicans* virulence

**Fig 7:**
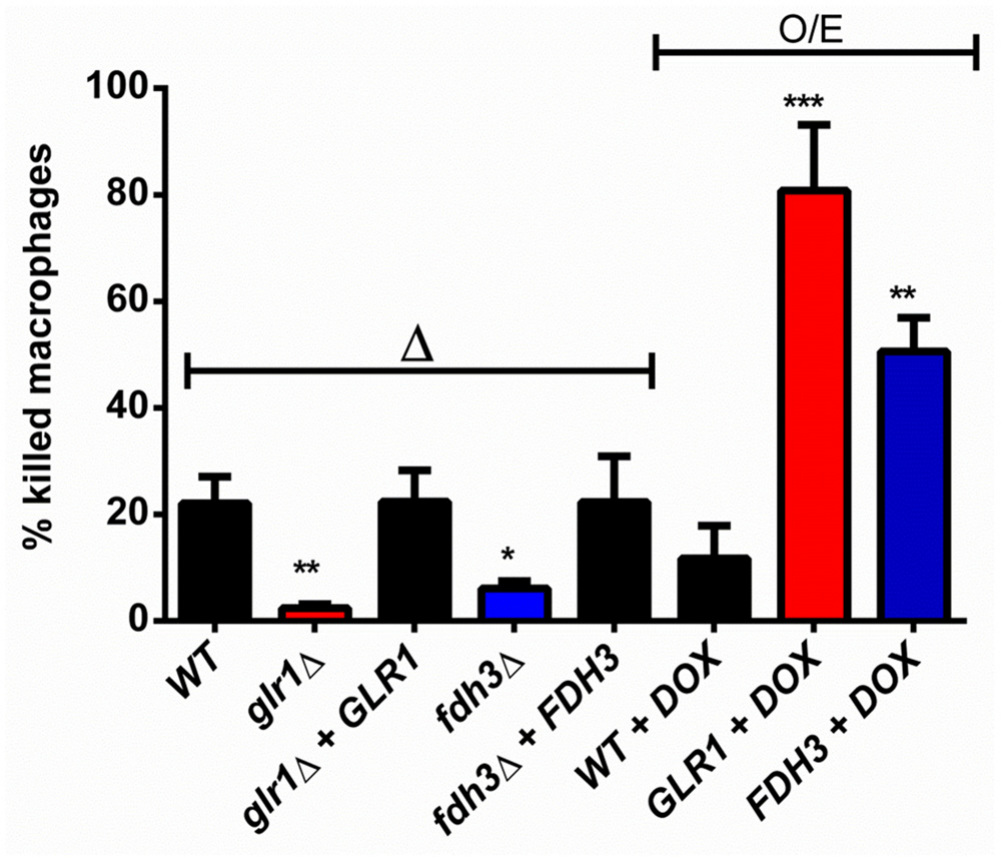
Deletion or overexpression of *GLR1* or *FDH3* alters the ability of *C*. *albicans* to kill macrophages. *C*. *albicans deletion* (Δ) and overexpression (O/E) mutants (1x10^6^ cells) were co-incubated with RAW264.7 macrophages (2x10^5^) for 3 h. The proportion of killed macrophages was determined following trypan blue staining: wild type (CPK05), *glr1*Δ (CKS10), *glr1*Δ+*GLR1* (CKS31), *fdh3*Δ (ATT1); *fdh3*Δ+*FDH3* (ATT4); WT+DOX, *tetON-empty* (CAMY203); GLR1+DOX, *tetON-GLR1* (ATT6); FDH3+DOX, *tetON-FDH3* (ATT7).

The above data support the hypothesis that Glr1 is a glutathione reductase playing a critical role in the detoxification of GSSG and maintenance of redox homeostasis in *C*. *albicans*, and that Fdh3 is a bi-functional enzyme playing critical roles in detoxification of GSNO and formaldehyde (Fig 2). Given the importance of oxidative and nitrosative stress in *C*. *albicans-*phagocyte interactions during infection [45,46], we reasoned that changes in *GLR1* and *FDH3* functionality might affect the ability of *C*. *albicans* cells to kill phagocytes. To test this, we measured the percentage of cultured murine macrophages (RAW264.7 cells) that were killed by wild type and mutant *C*. *albicans* cells. The *glr1*Δ and *fdh3*Δ mutants displayed significantly reduced ability to kill macrophages when compared to the wild type and reintegrant controls (Fig 7). Furthermore, doxycycline-dependent overexpression of *GLR1* or *FDH3* from the *tetON* promoter [47,48] increased the ability of *C*. *albicans* to kill macrophages (Fig 7), when compared to the parental strain. We conclude that the functions of Glr1 and Fdh3, and their contribution to the maintenance of redox homeostasis, are integral to *C*. *albicans* viability and potency during fungus-phagocyte interactions.

**Fig 8:**
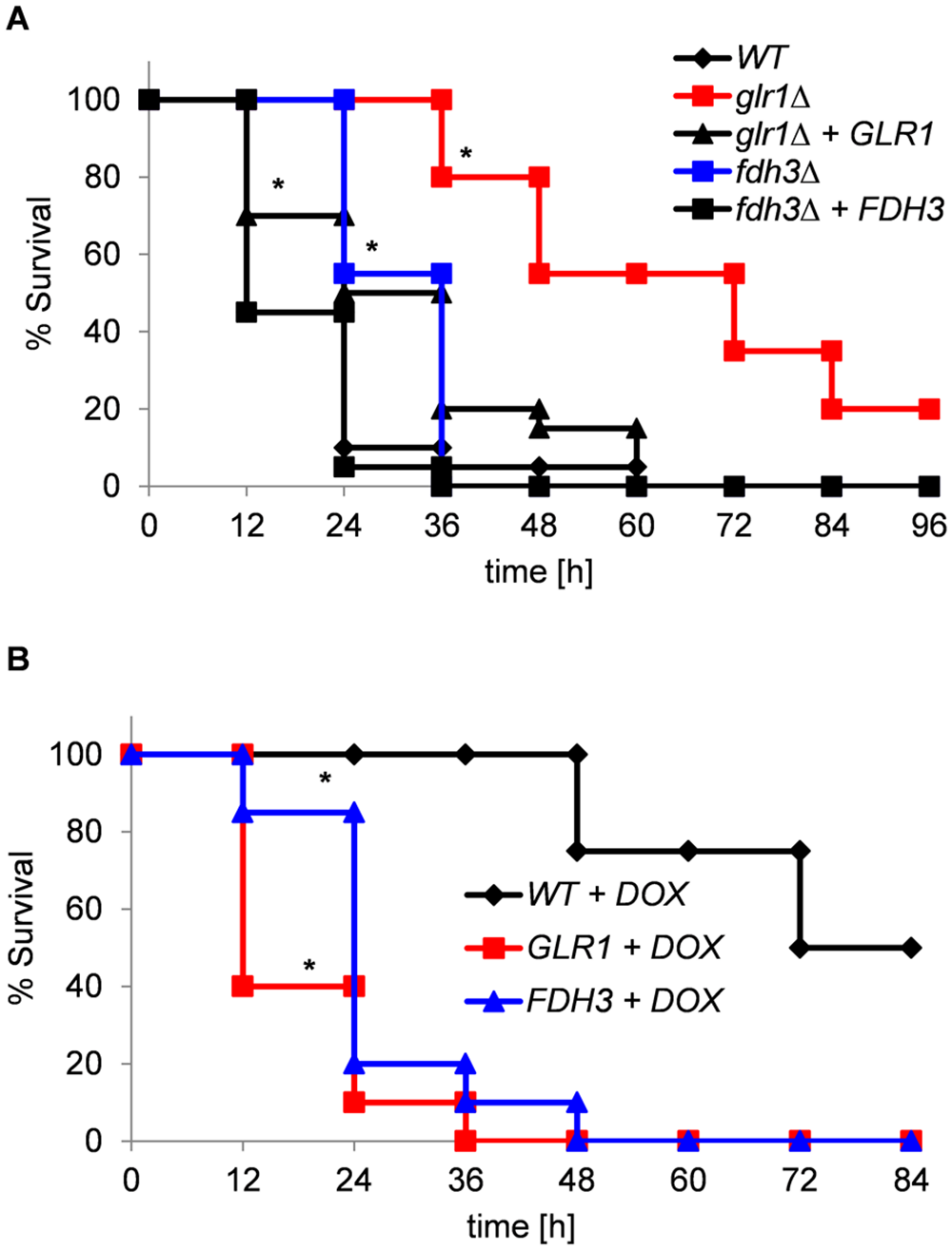
Virulence of *C*. *albicans GLR1* and *FDH3* mutants in the *G*. *mellonella* infection model. Kaplan-Meier plots of *G*. *mellonella* survival after injection with *C*. *albicans*. **(A)** Analysis of deletion mutants using a dose of 2.5x10^5^ *C*. *albicans* cells/larva: wild type (CPK05); *glr1*Δ (CKS10); *glr1*Δ+GLR1 (CKS31); *fdh3*Δ (ATT1); *fdh3*Δ+*FDH3* (ATT4) (Table 1). **(B)** Analysis of overexpression mutants using a lower dose of 1x10^4^ *C*. *albicans* cells/larva: WT+DOX, *tetON-empty* (CAMY203); GLR1+DOX, *tetON-GLR1* (ATT6); FDH3+DOX, *tetON-FDH3* (ATT7).

Based on their effects during fungus-phagocyte interactions, it was conceivable that *GLR1* and *FDH3* might influence the virulence of *C*. *albicans* during systemic infection. This was first tested in the invertebrate wax moth (*Galleria mellonella*) larval infection model, which has been established as a suitable proxy for systemic infection of the mammalian host [49]. Wild type *C*. *albicans* cells killed more than 90% of larvae within two days (Fig 8A). The *fdh3*Δ cells displayed a small, but reproducible, statistically significant reduction in virulence, whilst deletion of *GLR1* resulted in a much greater effect on virulence (Fig 8A). Doxycycline-induced overexpression of *GLR1* and *FDH3* dramatically increased the virulence of *C*. *albicans* (Fig 8B). Experiments utilising the overexpression mutants was performed with a reduced *C*. *albicans* inoculum to permit resolution of the increased virulence (1 x 10^4^ cells versus 2.5 x 10^5^ cells). These infection data are consistent with the macrophage killing assays (Fig 7) and show that both Glr1 and Fdh3 contribute to the virulence of *C*. *albicans*.

**Fig 9:**
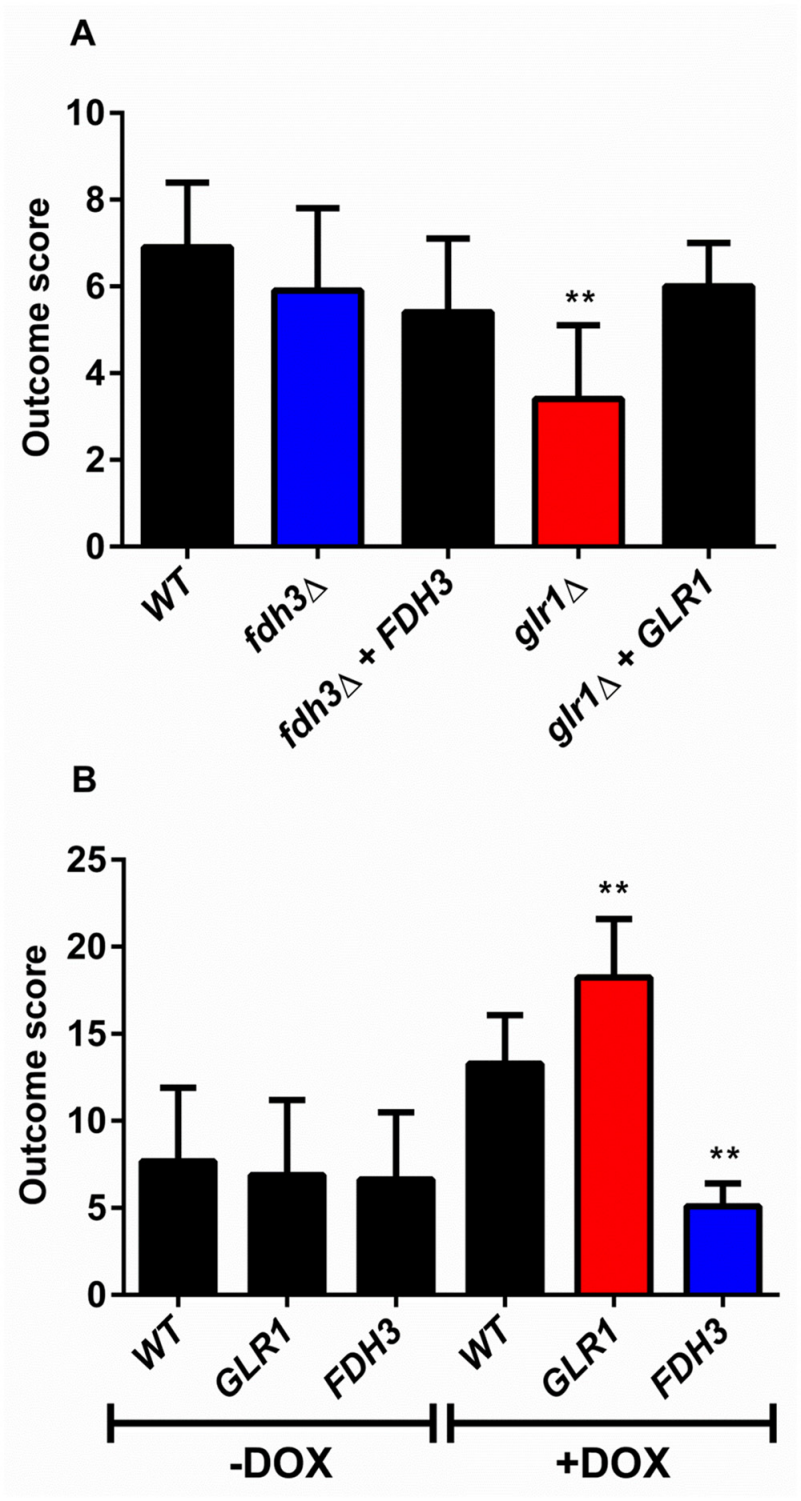
Virulence of *C*. *albicans GLR1* and *FDH3* mutants in the murine model of systemic candidiasis. Mice were infected with *C*. *albicans* strains by lateral tail vein injection, and infection outcome scores calculated after 72 h (Materials and Methods: means ± SEM; *n* = 6). **(A)** Analysis of deletion mutants: wild type (CPK05); *glr1*Δ (CKS10); *glr1*Δ+GLR1 (CKS31); *fdh3*Δ (ATT1); *fdh3*Δ+*FDH3* (ATT4). **(B)** Analysis of overexpression mutants in mice +/- doxycycline (DOX) in their drinking water: WT, *tetON-empty* (CAMY203); GLR1, *tetON-GLR1* (ATT6); FDH3, *tetON-FDH3* (ATT7).

Having established the impact of *GLR1* and *FDH3* on *C*. *albicans* virulence in the *G*. *mellonella* infection model, we then progressed to a mammalian model of systemic candidiasis. The virulence of the mutants was assayed using a three-day murine intravenous challenge model [50], which allows calculation of an outcome score. In this model, *fdh3*Δ cells displayed no significant difference in outcome score compared to the wild type and reintegrant controls (Fig 9A). However, the *glr1*Δ mutant showed a significant reduction in virulence. It is worth noting that Fdh3 inactivation had less of an impact than Glr1 inactivation upon virulence in the *G*. *mellonella* model (Fig 8A). This subtle effect might not have been detectable in the mouse experiments (Fig 9A).

We then examined the virulence of the *GLR1* and *FDH3* overexpression strains. We noted that the treatment of animals with doxycycline, which was required to induce *GLR1* and *FDH3* overexpression, resulted in increased outcome score for infection with the wild type *C*. *albicans* strain. Therefore, the virulence of the test strains was compared to this control (WT + DOX: Fig 9B). Once again, *GLR1* overexpression resulted in increased virulence of *C*. *albicans*. However, for reasons that are not clear, *FDH3* overexpression attenuated fungal virulence in this murine model of systemic infection. Whatever the molecular basis for this observation, it is clear that *GLR1* and *FDH3* functionality influences the virulence of *C*. *albicans* during systemic infection.

## Discussion

Numerous studies support the view that robust oxidative stress responses contribute to the pathogenicity of the major fungal pathogen, *C*. *albicans*. For example, inactivation of certain regulators that contribute to oxidative stress adaptation, such as the AP-1-like transcription factor Cap1 and the stress activated protein kinase Hog1, affect *C*. *albicans* virulence, albeit only slightly in the case of Cap1 [51–53]. Key enzymes involved in ROS detoxification, such as catalase (Cat1) and superoxide dismutase (Sod1, Sod5), are also essential for full virulence [13,54–56]. In addition, *C*. *albicans* mutants lacking certain components of the glutaredoxin (Gcs1, Grx2) and thioredoxin systems (Trx1) or mitochondrial-associated stress functions (Goa1) display attenuated virulence [31,57–59]. Therefore, we reasoned that key enzymes involved in the detoxification and recycling of glutathione adducts formed during oxidative stress are likely to be important for the maintenance of redox homeostasis in *C*. *albicans* and its virulence. When this work was initiated, we predicted that two annotated *C*. *albicans* genes might play key roles in GSSG and GSNO recycling. The first was Fdh3, a putative glutathione-dependent S-nitrosoglutathione reductase (GSNOR), which catalyses the generation of GSSG during the recycling of GSNO adducts. The second was a putative glutathione reductase (GR), Glr1, which was predicted to regenerate GSH from GSSG in an NADPH-dependent manner. Our bioinformatics analyses of *C*. *albicans* Glr1 and Fdh3 indicated that they are members of phylogenetically distinct GR and GSNOR families (Fig 1, and S1 and S2 Figs). We also noted that Fdh3 is related to bi-functional enzymes that also have formaldehyde dehydrogenase activity. Therefore we predicted that these highly conserved enzymes play differential but key roles in GSSG and GSNO recycling and formaldehyde detoxification (Fig 2), and in the maintenance of the redox homeostasis. Hence we reasoned that Glr1 and Fdh3 are probably crucial for *C*. *albicans* survival during host-pathogen interaction.

To test this, we generated *C*. *albicans glr1*Δ and *fdh3*Δ null mutants and compared their phenotypes to those of wild type and reintegrant control strains. Three key observations have confirmed our predictions. Firstly, *glr1*Δ cells are sensitive to oxidative stress, but not to nitrosative or formaldehyde stress. *Fdh3*Δ cells on the other hand display sensitivity to nitrosative or formaldehyde stress, but not to oxidative stress (Fig 3). Secondly, deletion of Glr1 completely blocks GSSG detoxification, and Fdh3 deletion slows GSNO detoxification (Fig 6). Thirdly, the glutathione redox potential is significantly perturbed in *glr1*Δ cells (Fig 6C).

The functions of Glr1 and Fdh3 are intimately linked, for example through their recycling of glutathione adducts, and in particular by the generation of GSSG by Fdh3-mediated recycling of GSNO (Fig 2). This linkage is reflected in several observations. For example, compensatory changes in *GLR1* and *FDH3* gene expression occur in *fdh3*Δ and *glr1*Δ cells, respectively (Figs 3 and 4). The increased *FDH3* expression in *glr1*Δ cells appears to result in elevated resistance to nitrosative stress (Fig 3C). Also, deletion of Glr1 leads to the induction of oxidative stress genes (*TRX1*) and redox-dependent protein folding functions (*PDI1*) in response to formaldehyde stress (Fig 5), presumably due to the excessive accumulation of GSSG under these conditions. Furthermore, deletion of Fdh3 slows GSSG detoxification (Fig 6A), presumably because *GLR1* expression is reduced approximately 4-fold in *fdh3*Δ cells (Fig 4).

Fdh3 does not appear to be the only enzyme in *C*. *albicans* that has denitrosylation or trans-nitrosylation activity because GSNO detoxification is only partially inhibited in *fdh3*Δ cells (Fig 6B). Nitric oxide (NO) release and NO signalling are under tight regulatory control in eukaryotic cells [60]. GSNO can act as a cellular NO “reservoir” as it can release NO or act as a substrate for the transfer of NO to target cysteine residues via trans-nitrosylation [61]. Moreover, several types of eukaryotic enzymes in addition to GSNORs [62–64] have trans-nitrosylase or denitrosylase activity, including thioredoxin [65], thioredoxin reductase [66–68], superoxide dismutase 1 [69,70], haemoglobin [71] and protein-disulphide isomerase [72,73]. However, the potential roles of these systems in GSNO detoxification have not yet been defined in *C*. *albicans*. In contrast, the identity of the key NO detoxification enzyme in *C*. *albicans* has been determined. This role has been assigned to the haem oxygenase, Yhb1 [43,74,75].

Given their key roles in the maintenance of glutathione redox homeostasis, it was to be expected that Glr1 and Fdh3 influence fungus-phagocyte interactions and contribute to the virulence of *C*. *albicans*. The deletion of Glr1 or Fdh3 reduced the ability of *C*. *albicans* cells to kill macrophages, and overexpression of *GLR1* or *FDH3* increased the potency of *C*. *albicans* (Fig 7). Also, *glr1*Δ and *fdh3*Δ cells displayed reduced virulence in the invertebrate *G*. *mellonella* infection model, whilst *GLR1* and *FDH3* overexpressing cells were more virulent (Fig 8). Furthermore, in a mammalian model of systemic candidiasis, *glr1*Δ cells displayed attenuated virulence, and *GLR1* overexpressing strain showed increased virulence (Fig 9). However, the behaviour of *FDH3* mutants differed between the invertebrate and mammalian models, *fdh3*Δ cells displaying no significant reduction in virulence and *FDH3* overexpressing cells having reduced virulence.

Why might *fdh3*Δ and *FDH3* over-expressing cells display differences in their virulence phenotypes between the *G*. *mellonella* and murine infection models? A number of studies have shown that there is a good correlation between the outcome of insect and mouse infection models [76–78]. However, this might not always be the case. Possible differences in the NO concentrations experienced by *C*. *albicans* cells in these infection models might account for the differential impact of *FDH3* mutations. However, Bergin *et al*. (2005) have reported that the *G*. *mellonella* haemocytes act in a similar fashion to human neutrophils in that they are able to phagocytose bacteria and fungi and generate superoxide [79–81]. Alternatively, the differential virulence phenotypes for *FDH3* mutants might relate to the impact of *FDH3* upon *GLR1* expression (Figs 4 and 5). This might be significant in the context of the strong oxidising potential of mouse immune cells, and the complexity of mammalian niches [46].

Why might *fdh3*Δ cells, but not *glr1*Δ cells display contrasting phenotypes in the invertebrate and mammalian infection models? The basis for this probably lies in the observation that *glr1*Δ cells have a lower glutathione redox buffering capacity than *fdh3*Δ cells (Fig 6C). Therefore, *glr1*Δ cells are more vulnerable to ROS or RNS than *fdh3*Δ cells. Nevertheless, despite the complexities of *FDH3* virulence phenotypes, it is clear that *FHD3* and *GLR1* functionality strongly influences *C*. *albicans* pathogenicity.

To conclude, this study has demonstrated that Glr1 and Fdh3 play important roles in oxidative, nitrosative and formaldehyde stress response in *C*. *albicans*, and that this contributes to *C*. *albicans* pathogenicity. Our findings emphasise the importance of the glutathione system and the key role of enzymes involved in the maintenance of intracellular redox homeostasis during host infection.

## Materials and Methods

### Strains and growth conditions

*C*. *albicans* strains (Table 1) were grown at 30°C in YPDT (2% glucose, 2% Mycological peptone, 1% yeast extract, 100 mM Tris-HCl, pH 7.4) [82], or SD [83] supplemented with Complete Supplement (CSM) Drop-out mixture lacking uracil/uridine or arginine (Formedium). Nourseothricin was added to a final concentration of 200 μg/mL.

**Table 1:** *C*. *albicans* strains.

Name	Description	Genotype	Source
CAI4		*ura3Δ*::*imm434/Δura3Δ*::*imm434*	[[97–99]
CA372	CAI4 + CIp10	*ura3Δ*::*imm434/Δura3Δ*::*imm434*, *RPS1-Clp10 (URA3)*	[97,98]
RM1000		*ura3Δ*::*imm434/ura3Δ*::*imm434*, *his1Δ*::*hisG/his1Δ*::*hisG*	[100]
CA674	RM1000 + CIp20	*ura3Δ*::*imm434/Δura3Δ*::*imm434*, *his1Δ*::*hisG/his1Δ*::*hisG*, *RPS1-CIp20 (URA3*, *HIS1)*	[101]
BWP17		*ura3Δ*::*imm434/Δura3Δ*::*imm434*, *his1Δ*::*hisG/his1Δ*::*hisG*, *arg4Δ*::*hisG/arg4Δ*::*hisG*	[102]
CA1206	BWP17 + CIp30	*ura3Δ*::*imm434/Δura3Δ*::*imm434*, *his1Δ*::*hisG/his1Δ*::*hisG*, *arg4Δ*::*hisG/arg4Δ*::*hisG*, *RPS1- CIp30 (URA3*, *HIS1*, *ARG4)*	[59]
CPK05	prototroph	*ura3Δ*::*imm434/Δura3Δ*::*URA3*, *his1Δ*::*hisG/his1Δ*::*HIS1*, *arg4Δ*::*hisG/arg4Δ*::*ARG4*	[40]
CKS10	*GLR1/glr1*Δ	*ura3Δ*::*imm434/Δura3Δ*::*imm434*::*URA3*, *his1Δ*::*hisG/his1Δ*::*hisG*, *arg4Δ*::*hisG/arg4Δ*::*hisG*, *glr1Δ*::*HIS1/glr1Δ*::*ARG4*	[40]
CKS31	*glr1*Δ/*glr1*Δ +*GLR1*	*ura3Δ*::*imm434/Δura3Δ*::*imm434*::*URA3-GLR1*, *his1Δ*::*hisG/his1Δ*::*hisG*, *arg4Δ*::*hisG/arg4Δ*::*hisG*, *glr1Δ*::*ARG4/glr11Δ*::*HIS1*	[40]
ATT0	*FDH3/fdh3Δ*	*ura3Δ*::*imm434/Δura3Δ*::*imm434*, *his1Δ*::*hisG/his1Δ*::*hisG*, *arg4Δ*::*hisG/arg4Δ*::*hisG*, *FDH3/fdh3Δ*::*loxP-ARG4-loxP*	This study
ATT1	*fdh3Δ*/*fdh3Δ*	*ura3Δ*::*imm434/Δura3Δ*::*imm434*, *his1Δ*::*hisG/his1Δ*::*hisG*, *arg4Δ*::*hisG/arg4Δ*::*hisG*, *fdh3Δ*::*loxP-URA3-loxP/fdh3Δ*::*loxP-ARG4-loxP*	This study
ATT4	*fdh3Δ*/*fdh3Δ+FDH3*	*ura3Δ*::*imm434/Δura3Δ*::*imm434*, *his1Δ*::*hisG/his1Δ*::*hisG*, *arg4Δ*::*hisG/arg4Δ*::*hisG*, *fdh3Δ*::*loxP-URA3-loxP/fdh3Δ*::*loxP-ARG4-loxP*, *RPS1- CIp30-FDH3 (URA3*, *HIS1*, *ARG4)*	This study
CEC2175		*ura3Δ*::*imm434/Δura3Δ*::*imm434*, *his1Δ*::*hisG/his1Δ*::*hisG*, *arg4Δ*::*hisG/arg4Δ*::*hisG*, *ADH1/adh1*::*ADH1p-cartTA*::*SAT1*	[48]
CAMY203	CEC2175 *tetON-empty*	*ura3Δ*::*imm434/Δura3Δ*::*imm434*, *his1Δ*::*hisG/his1Δ*::*hisG*, *arg4Δ*::*hisG/arg4Δ*::*hisG*, *ADH1/adh1*::*ADH1p-cartTA*::*SAT1*, *RPS1/RPS1*::*CIp10-P*_*TET*_ *-GTW*	This study
ATT6	CEC2175 *tetON-GLR1*	*ura3Δ*::*imm434/Δura3Δ*::*imm434*, *his1Δ*::*hisG/his1Δ*::*hisG*, *arg4Δ*::*hisG/arg4Δ*::*hisG*, *ADH1/adh1*::*ADH1p-cartTA*::*SAT1*, *RPS1/RPS1*::*CIp10-P*_*TET*_ *-GLR1*	This study
ATT7	CEC2175 *tetON-FDH3*	*ura3Δ*::*imm434/Δura3Δ*::*imm434*, *his1Δ*::*hisG/his1Δ*::*hisG*, *arg4Δ*::*hisG/arg4Δ*::*hisG*, *ADH1/adh1*::*ADH1p-cartTA*::*SAT1*, *RPS1/RPS1*::*CIp10-P*_*TET*_ *-FDH3*	This study

### Stress conditions

To examine stress responses, *C*. *albicans* cells were grown overnight at 30°C and 200 rpm in YPDT. Cells were then re-inoculated into YPDT to an OD_600_ of 0.2 and grown to an OD_600_ of 0.8 at 30°C and 200 rpm. Cultures were then diluted four-fold in fresh YPDT, mixed with the appropriate stressors, and incubated at 30°C and 200 rpm. Stress was imposed using 1 M NaCl, 5 mM formaldehyde, 5 mM H_2_O_2_, 2.5 mM DPTA NONOate, or 2.5 mM freshly generated CysNO [82].

To examine oxidative and formaldehyde stress resistance, serial two-fold dilutions of *C*. *albicans* cells were plated onto YPD plates supplemented with 7.5 mM H_2_O_2_ or 5 mM formaldehyde. Plates were incubated for 48 h at 30°C. Results shown are representative of data from at least three independent experiments. Formaldehyde sensitivity was also tested by growth at 30°C in YPD containing a range of formaldehyde concentrations using 96 well microtiter plates. Plates were incubated with shaking at 30°C, and growth assessed relative to untreated controls by measuring the OD_620_ after 48 h. Experiments were performed in triplicate and one representative experiment was shown.

Nitrosative stress resistance was assessed by measuring adaptation times. Strains were grown overnight in 10 mL YPDT at 30°C, and diluted to an OD_600_ of 0.1 in fresh YPDT. Aliquots (100 μL) were added to 96 well microtiter plates (Costar) containing 100 μL YPDT containing 5 mM DPTA NONOate or CysNO (final concentration 2.5 mM). Plates were incubated with shaking at 30°C and the OD_620_ measured every 20 min for 48–72 h in a FluroStar Optima plate reader (BMG Labtech). Stress adaptation times were determined by measuring the inflection times for each growth curve, as described previously [84]. Data represent the means and standard deviations from at least three independent experiments.

To prepare CysNO, 4 mL of 0.34 mM L-cysteine, 0.75 M HCl was added to 5 mL of 0.5 mM NaNO_2_. After the solution turned deep red, 2.5 mL 1 M NaOH was added and the concentration of CysNO measured at 335 nm (ε_335nm_ 503 M^-1^cm^-1^) [85].

### Plasmid and strain construction

To generate *fdh3Δ* null mutants, the two *FDH3* alleles in *C*. *albicans* BWP17 (Table 1) were sequentially disrupted using *fdh3Δ*::*loxP-ARG4-loxP* and *fdh3Δ*::*loxP-URA3-loxP* markers [86] that were PCR amplified using the primers described in S1 Table. This generated the heterozygous mutant *fdh3Δ*::*LAL/FDH3* (ATT0) and then the homozygous *fdh3Δ*::*LAL/fdh3Δ*::LUL null mutant (ATT1) (Table 1). To construct the reintegrant control, the *FDH3* gene was PCR amplified (S1 Table), cloned into CIp30 [86,87], and the resulting CIp30-*FDH3* plasmid digested with *StuI* and integrated at the *RPS1* locus of *C*. *albicans* ATT1 to generate the *fdh3Δ/fdh3Δ*/*FDH3* reintegrant strain, ATT4 (Table 1). The genotype of these strains was confirmed by diagnostic PCR using primers described in S1 Table.

To construct *glr1Δ* null strains, *glr1Δ*::*ARG4* and *glr1Δ*::*HIS1* disruption cassettes were generated by PCR amplification (S1 Table) from plasmids pFA-HIS1 and pFA-ARG4 [11]. The disruption cassettes were transformed into BWP17 to generate a *glr1*Δ::*ARG4*/*glr1*Δ::*HIS1* strain. To generate the reintegrant control, the *GLR1* gene was PCR amplified (S1 Table) and cloned into pLUBP [88] to make pLUBP-GLR1 (*URA3*). The empty pLUBP and pLUBP-GLR1 plasmids were linearized with *XhoI/PacI* and transformed into the *glr1*Δ::*ARG4*/*glr1*Δ::*HIS1* to create the *glr1*Δ::*ARG4*/*glr1*0Δ::*HIS1 URA3* (CKS10) and *glr1*Δ::*ARG4*/*glr1*Δ::*HIS1 URA3-GLR1* strains (CKS31) (Table 1).

The doxycycline-conditional over-expression strains *tetON-GLR1* (ATT6) and *tetON-FDH3* (ATT7) were generated as described previously [48,89]. The CIp10-based *tetON* expression plasmids were linearized with *StuI*, and transformed into *C*. *albicans* CEC2175 (Table 1). The genotypes of these strains were confirmed by diagnostic PCR using the primers described in S1 Table.

### Transcript analyses

*C*. *albicans* cells were harvested, flash-frozen in liquid N_2,_ and RNA extracted as described previously [90,91]. RNA integrity was confirmed using Bioanalyzer RNA 6000 Nano Assay Protocol according to the manufacturer’s instructions (Agilent; Stockport, UK) [92]. cDNA was prepared using Superscript II as per the manufacturer’s protocols (Invitrogen Ltd.; Paisley, UK). Transcript levels were measured by qRT-PCR relative to the internal *ACT1* mRNA control using the primers listed in S1 Table with a LightCycler480 system (Roche Applied Science) using the Roche Universal Probe library [15]. Data represent the means and standard deviations from at least three independent experiments.

### GSH, GSSG and GSNO assays

*C*. *albicans* cells were harvested [82] and protein extracts prepared in 0.5 M citrate, pH 5.0) as GSNO was found to be unstable at pH 8.0. For GSNO assays, 5 μL of 1 mM NADH and 10 μL of 250 μM GSNO were added to 10 μL of protein extract, and samples were derivatized at 0, 2, 5, 10, 20 and 30 min. For GSSG assays, 5 μL of 1 mM NADPH and 10 μL of 250 μM GSSG were added to 10 μL of protein extract, and samples were derivatized at 0, 2, 5, 10, 20 and 30 min. The derivatization and analysis were performed as previously described [93] using a Thermo Surveyor LC system coupled to a TSQ Quantum, triple quadrupole mass spectrometer (Thermo Scientific, UK). The following SRM transitions were used for quantification; Glu-Glu (internal standard) *m/z* 277–241, GSNO *m/z* 337–307, GSNEM *m/z* 433–304 and GSSG *m/z* 613–355. Peak integration and quantification was performed using Xcalibur software (Version 2.0.7.SP2). GSH, GSSG and GSNO concentrations were then calculated relative to authentic standards. Data were normalised against total protein. Data represent the means and standard deviations from at least three independent experiments.

The redox potential was calculated using the GSH and GSSG concentrations calculated using the following equation based on the Nernst equation [20].

$$\Delta \text{E = -264mV-}\frac{\text{60.2}}{\text{2}}\text{log}\frac{{\left[GSH\right]}^{2}}{\left[\text{GSSG}\right]}\text{mV}$$

(30°C, pH 7.4)

### Macrophage killing assay

RAW264.7 murine macrophages were cultured in Dulbecco’s modified Eagle’s medium supplemented with 10% fetal calf serum. *C*. *albicans* cells (1x10^6^) were co-incubated with RAW264.7 macrophages (2x10^5^) for 3 h at 37°C. For doxycycline-induced over-expression, *C*. *albicans* cells were prepared by growing overnight in YPDT containing 100 μg/mL doxycycline. Wells were washed with PBS and stained with trypan blue. Macrophages were fixed in 3% formaldehyde for 3 min and images were recorded with an Zeiss Axio Observer Z1 inverted microscope and Zeiss Hrm camera. Images were analysed using Axiovision 4.8.2 software, and the percentage of killed macrophages calculated by analysing at least 200 macrophages per well [94]. All data were generated in triplicate for at least three independent experiments and one representative experiment was represented.

### *Galleria mellonella* virulence assay

The virulence of *C*. *albicans* strains was assessed using groups of 20 *G*. *mellonella* larvae (Livefoods, UK) in the sixth instar as described previously [95]. *C*. *albicans* inocula were grown in YPDT at 30°C overnight with agitation. For doxycycline-induced over-expression, 100 μg/mL doxycycline was added to the medium. *C*. *albicans* cells were washed three times in PBS, re-suspended in PBS at 2.5 x 10^5^ cells/mL and 10 μL of suspension injected via the last proleg [95]. Untouched larvae and larvae injected with PBS +/- doxycycline served as controls. After injection larvae were incubated at 37°C and the number of dead larvae scored every 12 h. Statistical analyses were performed using the Kaplan-Meier log rank test. Differences with a *P* value < 0.05 were considered significant. Three independent experiments were performed, and data from one representative experiment are presented.

### Murine virulence assay

The three-day murine intravenous challenge model of *C*. *albicans* infection [50] was used to determine the impact of *GLR1* and *FDH3* upon virulence. Female BALB/c mice (6–8 weeks old; Harlan, UK) were randomly assigned into groups of six mice, were housed in individually ventilated cages (IVCs) and were provided with food and drinking fluid *ad libitum*. Each group of mice was inoculated via the lateral tail vein with *C*. *albicans* cells (3 x 10^4^ CFU g^-1^ mouse body weight) grown at 30°C in NGY +/- 50 μg/mL doxycycline [96]. The fungal inoculum used for each mouse group was also randomised. For doxycycline treated mice, mice were either provided with 5% sucrose or 5% sucrose containing 2 mg/mL doxycycline as their drinking fluid. Mice were monitored and weighed daily. After 72 h, mice were weighed, terminated by cervical dislocation, and renal fungal burdens determined. The outcome score [50] was calculated based on the fungal kidney burdens and percentage weight change at 72 h. Statistical differences between body weight changes, kidney burdens and outcome scores were determined by Kruskall-Wallis and Mann-Whitney U tests using IBM SPSS (version 20).

### Ethics statement

All animal experimentation was performed under UK Home Office Project license 60/4135 and was approved by the UK Home Office and by the Animal Welfare and Ethical Review Body of the University of Aberdeen. All work conformed to European Directive 2010/63/EU.

Animals were carefully monitored for signs of distress during the infection studies. Distress was minimised by expert handling. Animals were weighed once daily, and monitored for changes in condition at least twice daily. Animals were euthanized humanely by cervical dislocation when they showed signs of severe illness, i.e. they had a ruffled coat, displayed a hunched posture, were unwilling to move around the cage and had lost 20% of their initial body weight. There were no unexpected incidents of mortality during this study. Analgesia and anaesthetics were not required.

### Statistical analyses

Unless otherwise stated, data are expressed as means plus standard deviations from at least three independent experiments: * *p*< 0.05; ** *p*<0.001; and *** *p* <0.0001. Statistical significance was determined by one-way ANOVA with post hoc analysis using Dunnett's t-tests with a 95% confidence level. Analyses were carried out using Prism 5.0 (Graphpad). Data are represented in means ± SD.

## Supporting Information

S1 Fig *Candida albicans GLR1* encodes a NADPH-dependent glutathione reductase. The sequence alignment of homologs of the NADPH-dependent glutathione reductase *GLR1* (C5_01520C) was generated using ClustalW. The homologs from *Saccharomyces cerevisiae*, *Schizosaccharomyces pombe*, *Mus musculus*, *Homo sapiens* and *Caenorhabditis elegans* used for the multiple sequence alignments were obtained from NCBI/ BLAST are shown. The conserved NADH binding domain within a larger FAD bindingdomain and the C-terminal dimerisation domain are illustrated in red and green, respectively.
(TIF)

S2 Fig *Candida albicans FDH3* encodes a GSH-dependent formaldehyde dehydrogenase class III. The sequence alignment of homologs of the *Candida albicans* GSH-dependent formaldehyde dehydrogenase *FDH3* (CR_10250C_A) was generated using ClustalW. The homologs from *Saccharomyces cerevisiae*, *Schizosaccharomyces pombe*, *Mus musculus*, *Homo sapiens*, *Drosophila melanogaster* and *Caenorhabditis elegans* used for the multiple sequence alignments were obtained from NCBI/ BLAST are shown. The conserved catalytic domain of the alcohol dehydrogenases class III and the C-terminal cofactor-binding domain that reversibly binds NAD(H) are illustrated in red and green, respectively.
(TIF)

S1 Table PCR Primers. (DOCX)
