# Supplementary figures and images for: Contribution of Fdh3 and Glr1 to Glutathione Redox State, Stress Adaptation and Virulence in Candida albicans
Source: PLoS One. 2015 Jun 3;10(6):e0126940. doi: 10.1371/journal.pone.0126940 (PMC4454436; doi:10.1371/journal.pone.0126940)

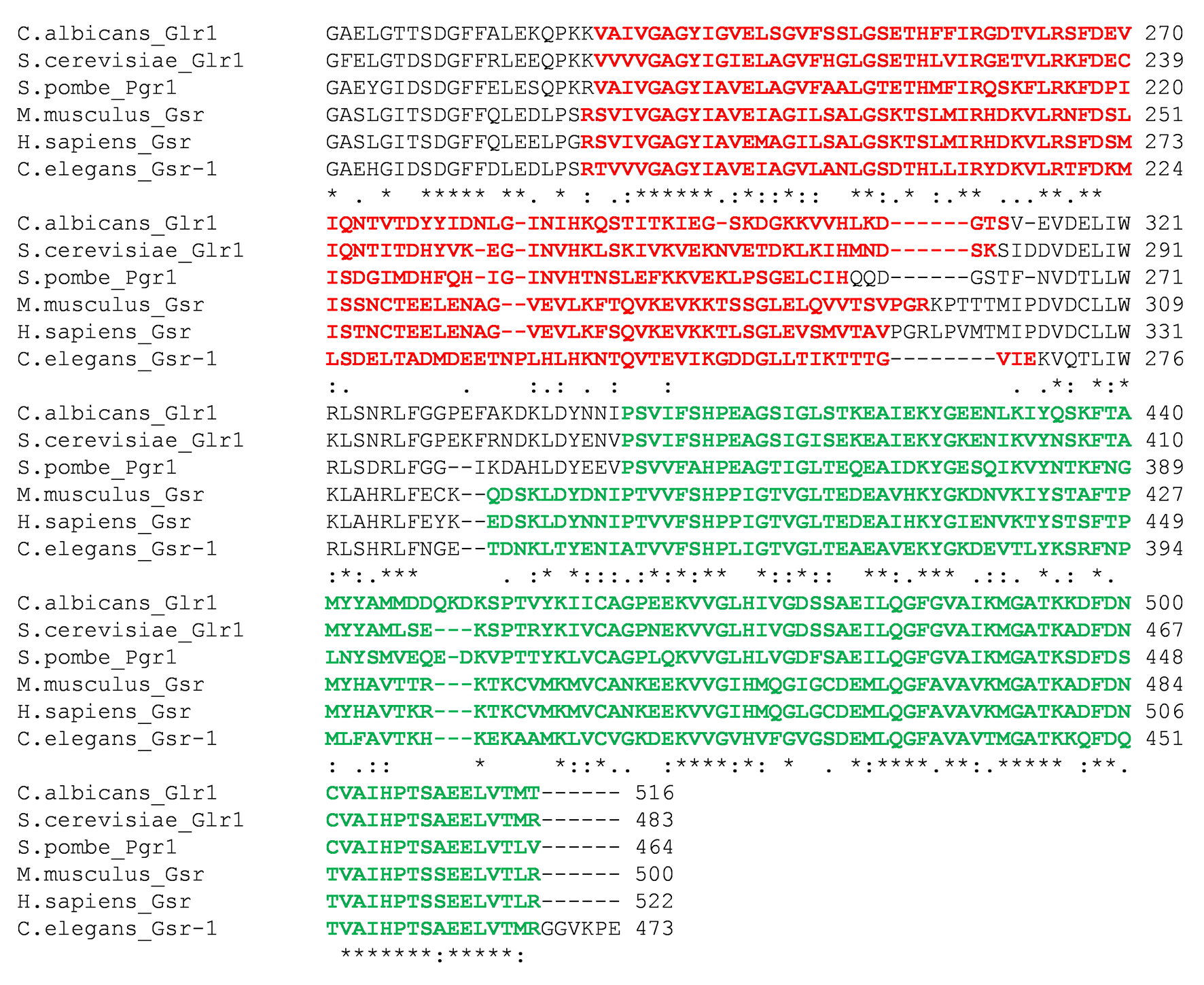

Supplement: S1 Fig — The sequence alignment of homologs of the NADPH-dependent glutathione reductase GLR1 (C5_01520C) was generated using ClustalW. The homologs from Saccharomyces cerevisiae, Schizosaccharomyces pombe, Mus musculus, Homo sapiens and Caenorhabditis elegans used for the multiple sequence alignments were obtained from NCBI/ BLAST are shown. The conserved NADH binding domain within a larger FAD bindingdomain and the C-terminal dimerisation domain are illustrated in red and green, respectively. (TIF) [file pone.0126940.s001.tif]

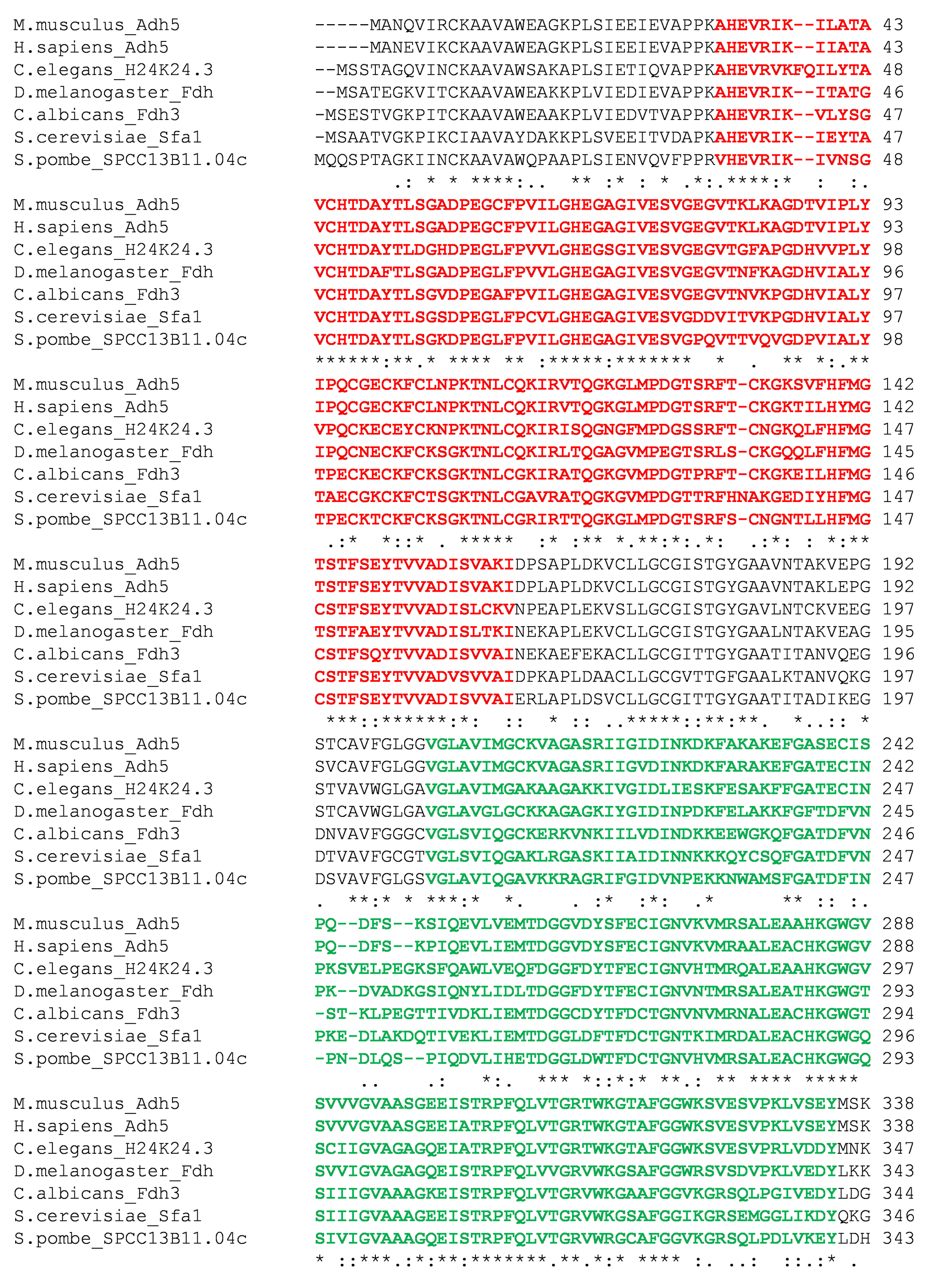

Supplement: S2 Fig — The sequence alignment of homologs of the Candida albicans GSH-dependent formaldehyde dehydrogenase FDH3 (CR_10250C_A) was generated using ClustalW. The homologs from Saccharomyces cerevisiae, Schizosaccharomyces pombe, Mus musculus, Homo sapiens, Drosophila melanogaster and Caenorhabditis elegans used for the multiple sequence alignments were obtained from NCBI/ BLAST are shown. The conserved catalytic domain of the alcohol dehydrogenases class III and the C-terminal cofactor-binding domain that reversibly binds NAD(H) are illustrated in red and green, respectively. (TIF) [file pone.0126940.s002.tif]
