# Supplementary material for: Contribution of Fdh3 and Glr1 to Glutathione Redox State, Stress Adaptation and Virulence in Candida albicans
Source: PLoS One. 2015 Jun 3;10(6):e0126940. doi: 10.1371/journal.pone.0126940 (PMC4454436; doi:10.1371/journal.pone.0126940)
Supplement: S1 Table — (DOCX) [file pone.0126940.s003.docx]

**Table S1. PCR Primers.**

| **Name** | **Sequence (5' - 3')** | **Application** |
| --- | --- | --- |
| F FDH3-LAL (P1) | AAAGATCGAGAAAACAAAAAAAAAGGGGAAATTTTACAATCTCTCTTTCTTACTTGTTCATTTTTATAATCAATTATATTTATAAATTAATATCACTACACCTCTTCGCTATTACGCCAG | Amplification of *fdh3::LAL* cassette |
| R FDH3-LAL (P2) | CCTTTATAGATAGTAAATATACCTCATATTAATATAAAAATAAATCCTATTAAAGATCTATACTTTGGTCCTAACTATCTTAAAGTTTAGGTGCTCCTTCGCAGATTACCCTGTTATCCCTA | Amplification of *fdh3-LAL* cassette |
| F FDH3-LUL2 (P3) | AAAGATCGAGAAAACAAAAAAAAAGGGGAAATTTTACAATCTCTCTTTCTTACTTGTTCATTTTTATAATCAATTATATTTATAAATTAATATCACTACACCAGGGTTTTCCCAGTCACG | Amplification of *fdh3-LUL2* cassette |
| R FDH3-LUL2 (P4) | CCTTTATAGATAGTAAATATACCTCATATTAATATAAAAATAAATCCTATTAAAGATCTATACTTTGGTCCTAACTATCTTAAAGTTTAGGTGCTCCTTCACTAAAGGGAACAAAAGC | Amplification of *fdh3-LUL2* cassette |
| F FDH3 dia (P5) | CGAAGATGTCACCGTTGCC | Diagnosis of *FDH3* |
| R FDH3 dia (P6) | CAGTAATGGTGGCCGCAC | Diagnosis of *FDH3* |
| F FDH3-LAL dia (P7) | GGTGGTCGTTTCACCGG | Diagnosis of *fdh3-LAL* allele |
| R FDH3-LAL/LUL2 (P8) | CTATCTTAAAGTTTAGGTGCTCCTTC | Diagnosis of *fdh3-LAL* and *fdh3-LUL2* alleles |
| F FDH3-LUL2 dia (P9) | GGTTCAATAGTGGATTCATAGG | Diagnosis of *fdh3-LUL2* allele |
| F FDH3-Reinte-SalI | GGAGAGgtcgacGTACAGTCTACCATCATATC | Amplification of *FDH3* reintegrant |
| R FDH3-Reinte-MluI | GGAGAGacgcgtGATCAAGCTTTATTAGAAAC | Amplification of *FDH3* reintegrant |
| F FDH3-CRE Probe | CGTATCGTTGCTGACC | Southern blotting for *FDH3* |
| R FDH3-CRE Probe | CCATCGATTGCTTTTATACCC | Southern blotting for *FDH3* |
| KS09 | GCTTCAATTTCTAACTTTTCAAGTTGCATTTATATTTTCATAAGTTTTTTTTTTCTTTTTGGTTCTTCTTTTTAGGAAGCTTCGTACGCTGCAGGTC | GLR1_S1 |
| KS10 | CACATAACTACGTACTTTCTATTGAAGAACCGATTAAGAACAATTGTTGTTGTTATCTAAAAAATAGGACTCATTCTGATATCATCGATGAATTCGAG | GLR1_S2 |
| KS12 | GCTTCAATTTCTAACTTTTCAAG | GLR1_F_Re |
| KS13 | CACATAACTACGTACTTTCTATTG | GLR1_R_Re |
| KS55 | CGGGATCCgaagaagtaaagcaaaatattttc | F_GLR1_compl_BamHI |
| KS56 | AAGGCCTcacataactacgtactttctattg | R_GLR1_compl_StuI |
| F ACT1 | ACCACCGGTATTGTTTTGGA | Primer with Probe #9 for qRT |
| R ACT1 | AGCGTAAATTGGAACAACGTG | Primer with Probe #9 for qRT |
| F GLR1 | AATTGGTGTTTTCCGCTGAC | Primer with Probe #59 for qRT |
| R GLR1 | TGAAGGAGGAACAATTGCTGT | Primer with Probe #59 for qRT |
| F FDH3 | AAGTGGTGTTGATCCAGAAGG | Primer with Probe #48 for qRT |
| R FDH3 | TTCACCGACACTTTCAACGA | Primer with Probe #48 for qRT |
| F TRX1 | ACCACACCAAGTGGCAAAA | Primer with Hyb *TRX1* ATT for qRT |
| R TRX1 | CGAATTCCAAACCCTTTTAAAGGAA | Primer with Hyb *TRX1* ATT for qRT |
| Hyb TRX1 ATT | 5’ FAM CGAATTCCAAACCCTTTTAAAGGAA 3’ TAMRA | qRT Probe for *TRX1* with 5' FAM and 3' TAMRA |
| CIp10-IS | GATATCGAATTCACGCGTAG | Diagnosis of CIp30 integration |
| CIp10-GS | GTACATTCCTACTCCGTTCG | Diagnosis of CIp30 integration |
| F O/E FDH3 | GGGGACAAGTTTGTACAAAAAAGCAGGCTtgATGTCTGAATCTACTGTTGGAAAGGTAAGT | Amplification of *FDH3* over-expression construct |
| R O/E FDH3 | GGGGACCACTTTGTACAAGAAAGCTGGGTcCCATAAAGAAACAACAGCTCTAATACAGTC | Amplification of *FDH3* over-expression construct |
| F O/E GLR1 | GGGGACAAGTTTGTACAAAAAAGCAGGCTtgATGTTTACTAATAGTATAATATCTAAATCA | Amplification of *GLR1* over-expression construct |
| R O/E GLR1 | GGGGACCACTTTGTACAAGAAAGCTGGGTcAGTCATTGTGACCAATTCTTCAGCTGATGT | Amplification of *GLR1* over-expression construct |
